# Practice nurse chlamydia testing in Australian general practice: a qualitative study of benefits, barriers and facilitators

**DOI:** 10.1186/s12875-015-0251-8

**Published:** 2015-03-14

**Authors:** Rebecca Lorch, Jane Hocking, Rebecca Guy, Alaina Vaisey, Anna Wood, Dyani Lewis, Meredith Temple-Smith, on behalf of the ACCEPt consortium

**Affiliations:** The Kirby Institute, University of New South Wales, Sydney, NSW Australia; Melbourne School of Population and Global Health, University of Melbourne, Melbourne, VIC Australia; Department of General Practice, University of Melbourne, Melbourne, VIC Australia

**Keywords:** Australia, Chlamydia, Clinical nursing research, General practice, Primary health care, Qualitative research

## Abstract

**Background:**

Chlamydia infection is a significant public health issue for young people; however, testing rates in Australian general practice are low. Practice nurses (PNs) could have an important role in contributing to increasing chlamydia testing rates. The Australian Chlamydia Control Effectiveness Pilot (ACCEPt), a large cluster randomised control trial of annual testing for 16 to 29 year olds in general practice, is the first to investigate the role of PNs in maximising testing rates. In order to assess the scope for PN involvement, we aimed to explore PN’s views in relation to involvement in chlamydia testing in general practice.

**Methods:**

Semi structured interviews were conducted between June 2011 and April 2012 with a purposive sample of 23 PNs participating in ACCEPt. Interview data was thematically analysed using a conventional content analysis approach.

**Results:**

The participants in our study supported an increased role for PNs in chlamydia testing and identified a number of patient benefits from this involvement, such as an improved service with greater access to testing and patients feeling more comfortable engaging with a nurse rather than a doctor. An alleviation of doctors’ workloads and expansion of the nurse’s role were also identified as benefits at a clinic level. Time and workload constraints were commonly considered barriers to chlamydia testing, along with concerns around privacy in the “small town” rural settings of the general practices. Some felt negative GP attitudes as well as issues with funding for PNs’ work could also be barriers. The provision of training and education, streamlining chlamydia testing pathways in clinics and changes to pathology ordering processes would facilitate nurse involvement in chlamydia testing.

**Conclusion:**

This study suggests that PNs could take a role in increasing chlamydia testing in general practice and that their involvement may result in possible benefits for patients, doctors, PNs and the community. Strategies to overcome identified barriers and facilitate their involvement must be further explored.

## Background

*Chlamydia trachomatis* (hereafter referred to as chlamydia) is a significant public health concern for young people. Chlamydia continues to be the most frequently reported notifiable condition in the United States (US), Europe and Australia. In 2012, around 1.4 million new chlamydia diagnoses were reported in the US, over 350,000 in 25 European Union member states and over 82 000 in Australia. The greatest burden of infection is in young people aged 15–29 years [1-3]. In young women, about 10% with untreated chlamydia will develop pelvic inflammatory disease [4], placing them at increased risk of serious reproductive morbidity including infertility and ectopic pregnancy [5,6]. Since most cases of chlamydia are asymptomatic [6], many infections go undetected.

A number of countries including the USA, Sweden, Denmark and New Zealand recommend yearly chlamydia screening in young adults; however, only England currently implements an organized chlamydia screening program [7,8], with screening programs planned for some European countries in the future [9]. An opportunistic program of chlamydia testing in general practice is currently being piloted – the Australian Chlamydia Control Effectiveness Pilot (ACCEPt) [10]. Over 85% of women and nearly two thirds of young men aged 16–29 years attend a general practice each year, making it an ideal setting for increased testing. Current Australian guidelines for general practitioners (GPs) recommend annual testing of this age group [11], but testing rates are low - 12.1% in women and 4.8% in men [12].

Practice nurses (PNs) are an integral part of general practice in Australia, the UK and other countries. With increasing pressures being placed on GPs’ time and workload, PNs could play an important role in contributing to increasing chlamydia testing rates. PNs have a well-established role in sexual health in countries such as the UK [13], but studies specifically examining PN involvement in chlamydia testing and management are scarce. A pilot study of chlamydia testing in general practice in New Zealand reported an increase in chlamydia testing when led by PNs [8,14] and a PN-led partner notification strategy in the UK was found to have similar effectiveness and costs to referral to a specialist health adviser [15]. To our knowledge, this is the first qualitative study exploring chlamydia testing and the role of PNs specifically in general practice. We aimed to explore PN’s views and opinions in relation to involvement in chlamydia testing in general practice.

## Methods

### Setting

This study was undertaken as part of ACCEPt. ACCEPt is the world’s first cluster randomised controlled trial (RCT) of an organised programme of yearly chlamydia testing in general practice and aims to determine whether annual chlamydia testing of 16–29 year old men and women can reduce the prevalence of chlamydia. ACCEPt is being conducted in 54 rural areas of four Australian states (Victoria, New South Wales, Queensland and South Australia). The risk of contamination between control and intervention sites was minimised by conducting ACCEPt in rural and regional areas and making the unit of randomisation the town, or postcode. By recruiting every clinic within a rural area, the possibility of patients attending non-participating in clinics was reduced. Towns were eligible to participate if they had a minimum population of 500 16–29 year olds, were at least 150 kilometers away from a capital city and had up to seven general practice clinics. Clinics varied considerably in size, with some solo GP-based clinics through to large clinics with over 20 GPs on staff.

A total of 143 general practice clinics have been enrolled and randomised to either receive a multifaceted intervention designed to facilitate increased testing or to continue with usual care. ACCEPt practices in towns randomised to the intervention arm could choose for PNs to become more involved in testing and management and receive PN specific education and training, plus financial incentives payable to the clinic. ACCEPt is the first large-scale chlamydia testing RCT to investigate the potential role of the PN in chlamydia testing.

Sexual and reproductive health in rural areas of Australia is largely provided by general practice as there are few specialist sexual health or family planning clinics available. General practice clinics (hereafter referred to as practices) in Australia are small businesses that receive most of their income via “fee per service”, with ~85% directly billed to the Australian government through the Medicare rebate system [16]. Until recently practices were also able to receive rebates for PN involvement in a narrow range of specific duties including immunisations, Pap smears and wound dressings. PNs’ work was thus often focused on and influenced by these income generating areas [17]. However, recent restructuring of PN funding under the Practice Nurse Incentive Program (PNIP), sees most task specific funding now replaced with a single funding stream. This change aims to support an enhanced role for PNs, allowing them more flexibility to undertake a broader range of activities in areas including preventative health [18] and aligning their role more closely to PNs in the UK [17].

### Sample

In-depth telephone interviews were conducted at the beginning of the trial and prior to randomisation with PNs employed in 23 different rural practices participating in ACCEPt. Purposive sampling [19] was used to maximize diversity and to provide the broadest representation of PNs. Selection was based on a number of characteristics that may influence chlamydia testing rates - size of clinic from data collected during clinic recruitment into ACCEPt; remoteness of area based on Australian Bureau of Statistics remoteness classification [20] and the PNs’ prior experience in chlamydia testing, as reported in cross sectional surveys completed by the PNs during ACCEPt recruitment. PNs were contacted by telephone by one of the authors (RL), and oral consent obtained for a telephone interview to be conducted at a later date.

### Interview schedule

A semi structured interview guide was drafted, reviewed by ACCEPt study staff and further refined. After the initial interviews ACCEPt study staff (RL, MTS) reviewed the transcripts to ensure comprehension of the questions, and quality of responses. Some minor changes were made to the interview guide following these initial interviews. The semi structured interviews covered the following domains: PNs’ current role in chlamydia testing and management, opinions on a PN role in chlamydia testing and management and perceived barriers and facilitators to this role. Participants also completed 12 structured questions capturing demographic, educational and employment history data (See Table 1).

**Table 1:** **Interview guide for ACCEPt baseline practice nurse interviews**

**Domain**	**Questions**
**Participant characteristics**	• Age and sex
	• Duration of nursing practice
	• Duration of employment in general practice
	• Employment status (Full/part time or casual)
	• Postgraduate qualifications
	• Education/training in sexual health
**Current involvement in sexual health/chlamydia testing**	• What is your role in relation to preventive health care with young men and women aged less than 30 years?
	• What is your involvement in the area of sexual health within the practice?
**Opinions on PN involvement in chlamydia testing**	• Can you tell me what you think about practice nurses taking an increased role in chlamydia testing in general practice?
	• What might be some of the benefits of practice nurses taking an increased role in chlamydia testing?
**Barriers and facilitators to PN involvement in testing**	• What could make it difficult for practice nurses to take an increased role in chlamydia testing?
	• What would make it easier for practice nurses to take an increased role in chlamydia testing?

### Interview procedure

The interviews were undertaken by one of the authors (RL), an ACCEPt research officer with a background in nursing and midwifery practice both in Australia and the UK. Close supervision and support was provided by MTS, a qualitative researcher with extensive experience in the area of sexual health and general practice. Interviews were conducted via telephone, recorded and transcribed verbatim. Interviews were undertaken until no new themes or insights were observed to be emerging i.e. data saturation was achieved [19].

### Analysis

NVivo qualitative data analysis software (QSR International Pty Ltd. Version 10, 2012) was used to organize and code the interview data. To ensure confidentiality during analysis, interview participants were assigned and identified by numbers prior to interview data being transferred into the data analysis software. Conventional content [21] and thematic analysis was undertaken, with interim analysis of early interviews occurring whilst the others were ongoing, to allow inclusion of unanticipated themes as prompts in subsequent interviews. After multiple readings, the transcripts were initially coded using a list of broad themes derived from the main categories of the interview schedule. Blocking, grouping and labelling of data was followed by secondary analysis to identify emerging themes. Analyst triangulation was utilised; all interviews were coded by one author (RL) and a sub-set of 12 interviews was coded separately by MTS. Consensus on both codes and themes was reached following comparison of analysis.

### Ethical approval

ACCEPt received ethical approval from the Royal Australian College of General Practitioners National Research and Evaluation Ethics Committee, the Aboriginal Health and Medical Research Council Ethics Committee and the University of Melbourne Human Research Ethics Committee.

## Results

A total of 23 interviews with PNs were completed between June 2011 and April 2012. The majority of the participants were female, aged 30–59 years and had been working in general practice for less than 10 years (See Table 2 for participant characteristics).

**Table 2:** **Participant characteristics (n = 23)**

**Characteristic**	**n (%)**
**Sex**	
Female	22 (96)
Male	1 (4)
**Age group (years)**	
30–44	10 (43)
45+	12 (53)
>60	1 (4)
**Location of general practice**	
New South Wales	10 (44)
Victoria	7 (30)
Queensland	4 (17)
South Australia	2 (9)
**Size of practice**	
Small (<3 GPs)	6 (26)
Medium (3–5 GPs)	9 (39)
Large (>5 GPs)	8 (35)
**Years since qualification**	
<15	6 (26)
15–29	13 (57)
30 -45	4 (17)
**Years working in any general practice**	
<5	13 (56)
5–10	8 (35)
11-20	2 (9)
**Past women’s health training**	
Yes	14 (61)
**Past sexual health training**	
Yes	6 (26)
**Chlamydia testing experience**	
Yes	12 (51)

The themes and sub-themes that arose from the interviews can be viewed in Table 3.

**Table 3:** **Themes/sub-themes arising from ACCEPt baseline practice nurse interviews**

**Domain**	**Themes**
**PNs current role in sexual health/chlamydia testing**	Advice and discussion
	Referral to/specimen collection for GP
	Complete consultation – women’s health
	Testing all young people as normal practice
**Opinion around PN involvement in chlamydia testing**	Support
	PNs suitability for role
**Benefits of PN involvement in chlamydia testing**	Patient benefits:
	• Increased access to testing
	• Patients prefer PNs
	• Patient empowerment
	GP benefits:
	• Ease workload
	PN benefits:
	• Role expansion – job satisfaction
**Barriers to PN involvement in chlamydia testing**	Time and workload
	Small town concerns:
	• Privacy and confidentiality
	GP attitudes:
	• Role conflict and handing over power
	Pathology ordering:
	• GP involvement and nurse autonomy
	Remuneration:
	• General practice as a business
	• Revenue attracting work
**Facilitators to PN involvement in chlamydia testing**	Education and training:
	• Knowledge and skills acquisition
	• Confidence and empowerment
	Change to pathology ordering
	Organisation of chlamydia testing:
	• Testing pathways
	Funding for PN chlamydia testing:
	• Item numbers for PN testing and PNIP

### Current role – chlamydia testing and management

Most of the PNs were involved at some level with chlamydia testing and management at their practices. Many provided safe sex advice and discussed sexual health issues with patients whilst collecting specimens for GPs or prior to referring to doctors for chlamydia testing.

*“The doctor might have ordered a series of tests for chlamydia and HIV and all those sorts of things. So obviously there has been an issue…I ask them do they practice safe sex. I will often have discussions with young people about making sure that they are trying to do the right thing.”* PN22

*“Basically it is an advisory role as much as anything. I don’t have obviously any prescribing role or anything like that or I can’t order pathology and stuff, so if they had unprotected then I suggest that they consider seeing the doctor.“*PN16

Of the PNs who initiated *and* carried out testing, most were doing so during Pap smear consultations and thus testing mostly women and very small numbers of young men. However, one of these PNs described how she sometimes saw both sexes:

*“Within the realms of just offering Pap smears and breast checks here I have also been able to address sexual health and offer chlamydia testing as well. Often I am the first port of call and for the females sometimes they say, “Can I bring my partner in as well?” So therefore I have had the chance to talk to both.”* PN12

Very few nurses reported the widespread routine testing of young people, which was described by one nurse:

***“****Anyone that is sexually active that comes into our clinic we recommend a chlamydia screen, an STI overall screen. And we just get them to do a urine sample and nearly everyone is willing to do it. We have a pretty good success rate in doing the screening.”* PN6

A number of PNs also reported some involvement in partner notification, although this was seen as a role of the GP by some.

*“I like the idea of the doctor actually speaking to someone and saying, “Right you need to think about your partners in this one as well.” So we leave it to the doctors.”* PN9

### Increased PN role in chlamydia testing and management

The majority of participants were supportive of having increased involvement in chlamydia testing and management, feeling PNs were suited to the role.

*“I am all for it. I think that we are often the first person the patient sees especially in that age group and we have an ideal opportunity to offer that.”* PN18

*“Yeah I think it is a great idea…Well I think in general nurses are a little bit more in tune with primary health care and preventative health”* PN25

### Benefits of PN involvement

#### Patient benefits

The PNs felt that their involvement in testing could lead to increased access to testing, diagnosis and treatment for young people, thus benefitting the community.

*“Hopefully more (tests) actually being done for young people …patients can access practice nurses more easily and freely.”* PN1

*“We are not even touching on it here; it is generally not bought up in a conversation with a doctor in a consultation… so I think if it we could make it more available then there would be a lot more testing.”* PN12

*“Well definitely it would benefit the community, (we) would be able to pick up chlamydia earlier, inform people earlier about it and information like that would be of obvious benefit.”* PN20

A number of PNs felt that patients preferred a consultation with the PN as they were more approachable and easier to talk to than the GP, especially for females.

*“Look I think young people feel a lot more comfortable speaking to a nurse…I think they feel a lot more at ease.”* PN6

*“I think it is better for practice nurses to be doing that, the one-on-one situation, the confidentiality is there and with the women I think they feel more comfortable speaking to another woman rather than to males.”* PN19

One nurse felt her interactions with the patients empowered them to feel more confident to discuss sexual health matters with the GP.

*“…sometimes if they go to see a doctor without seeing me, the doctor will just discuss with them the doctor’s preference not the patient’s preference…So by seeing me, having the information, they can ask questions and then this encourages the doctors to be more open.”* PN12

#### GP benefits

The majority of PNs thought that their involvement could benefit GPs by easing their workload and that chlamydia testing was an easy role to hand over to PNs, who may have more time to provide patient education.

*“It certainly takes the emphasis off the doctors having to do things like that. It is not a difficult test so if somebody else can do it for them I think that would be beneficial.”* PN19

*“Well I think a practice nurse can have a little bit more time (than doctors) therefore a bit more time with the education and answering questions if required.”* PN4

#### PN benefits

Finally, some felt that PNs themselves could benefit, with job satisfaction resulting from role expansion into chlamydia testing

*“And I guess I love doing anything new so anything that adds interest to your job too is good and keeps you a bit on your toes and keeps you a bit more current.”* PN21

### Barriers to an increased PN role in chlamydia testing

#### Time and workload

Time and workload constraints were commonly raised as an important barrier by many in our sample. At busy times chlamydia testing would take a lower priority than acute cases.

*“…time constraints for practice nurses. Their workload is pretty heavy and pretty varied too…And certainly you have to prioritise what you can do. If you have got chest pain it is a priority over asking someone about chlamydia.”* PN9

However, the barrier was not insurmountable for some.

*“There is really no barrier other than a time barrier…Our own time constraints are fairly big…we never have time to scratch ourselves so from that point of view that is a definite constraint. As far as it is us fitting it into our days which could be a problem, there would be a way around it.”* PN16

#### “Small town” concerns

Many nurses spoke about the experience of living in a “small town”, linking this to patients’ and their own concerns about confidentiality and testing.

*“…it is a small town and you sort of know everybody but you try and explain to patients about confidentiality but I suppose it is still in the back of their mind.”* PN14

*“One of the biggest barriers I guess for this town is that is a really small tight knit community. I have children the same age; my kids went to the school here so they are the same age as the guys that are young coming in here all the time…So as far as I am concerned I guess they might feel a bit uncomfortable I guess.”* PN8

#### GP attitudes

A number of PNs felt that GP attitudes towards PN involvement in testing could act as a barrier, with issues such as role conflict and GP concerns about handing over “power” to PNs identified.

*“When I worked in other clinics there were a lot of issues with me having that knowledge and me being able to do that. It is a case of ‘that is our role’, but then (the doctors) didn’t want to do it either. The doctors are just threatened I think, threatened that you will take over a role that they can do. If you are working with doctors who treat you as a hand maiden it is very difficult then to come forward and say, ‘Well actually I think (PNs) can do this or this’.”* PN8

*“I actually think there is a role for practice nurses in that and I suppose the one difficulty might be how doctors feel about it…Oh you know sometimes depending who you work for some of them like to be the ones to instigate things. … GP Management Plans, ‘well just don’t do it, let me see them first and if I decide their GP Management Plan then I will let you know to do it’. Do you know what I mean?”* PN22

*“We are realising that we can do more and more and offer more that complements the doctors but (we need to) bridge that understanding that we are not in competition with them”* PN12

#### Pathology ordering

The ordering of pathology for chlamydia testing was identified as a barrier by a number of PNs. In Australia federal legislation requires that when diagnostic pathology investigations attract a charge against Medicare (as is the case in general practice) the request form must be signed by a medical practitioner *before* the service is provided [22], requiring direct input from a GP who is often too busy to do this or uncomfortable with the nurse managing the process independently.

*“I think one of the things is addressing the pathology side of things. Like at this stage most doctors would want to be involved if there is any discussion on chlamydia. They wouldn’t feel comfortable just handing that straight over to the nurses. At this stage I think the doctors would be, from what I have seen, reluctant to let us have that sort of freedom.”* PN12

*“They have to be seen by a doctor to order the pathology and generally our doctors are sort of they are reasonably booked up on a day to day basis.”* PN18

#### Remuneration for PN testing

The issue of funding for PN activities was raised by some as an important barrier. These PNs felt that GPs run businesses and so it was desirable for PNs to be undertaking activities that attract revenue. Prior to the introduction of the PNIP (when some of these interviews took place), some nurse activities such as Pap smears were funded specifically, whereas chlamydia testing was not.

*“The principal, he is paying us good wages, well my boss does. He pays us good wages so if we are going to do more and more testing, this is my management hat coming back on now, if I am doing more and more testing for chlamydia he doesn’t get a cent to pay my wages.”* PN2

### Facilitators to an increased PN role in chlamydia testing

#### Education and training

By far, the most significant facilitator for the PNs was the provision of education and training, which would not only provide knowledge and skills, but empower the nurses in their role as chlamydia testers.

*“That would come, I think, with the education. And the knowledge and awareness to have the confidence to approach and talk to people about it.”* PN9

*“There is nothing more embarrassing than someone asks you a question and you can’t answer it. So it is important we are trained.”* PN13

*“I think nurses need to be educated and part of that is about increasing their self-esteem and their recognition that they have the power to make a difference with these things.”* PN3

#### Changes to pathology processes

Some PNs suggested changes to systems of pathology ordering as facilitators to increase PN involvement in testing.

*“Basically what we need is pre-signed forms from the doctor… if someone came and the moment arose then you could say, “Well look here is the form just slip it back to the pathology with the specimen of urine.” It just makes it very simple rather than having to involve a doctor to get that testing done”* PN12

#### Organisation of chlamydia testing

Changes to the organisation of chlamydia testing within practices could facilitate increased PN chlamydia testing, with strategies to streamline the process, set up “testing pathways” and include other staff suggested by some.

*“Doing chlamydia screens certainly isn’t a huge time spender so if we could find a way of trying to identify the young people that are coming into the clinic and try to capture them and give them a cup and a path form. It is fairly straight forward after that isn’t it?” PN6*

*“I have spoken to the doctors and they are happy …when those patients come through the girls at the front desk, we have to give them the responsibility of looking at their ages and they will then redirect them through to the nurses.”* PN13

### Funding for PN testing

A system for reimbursing the cost of PN involvement in chlamydia testing, such as a Medicare nurse item number, was identified as a facilitator by some nurses.

*“…so I don’t know is there anything that can be done like your Pap smear incentive number…because then that would then pay for some of the ongoing costs”* PN2

*“…it possibly helps us to be a little bit more pro-active some sort of incentive number payment I think…if you are busy you think, “Oh well I won’t do the chlamydia, I haven’t go time to talk about the chlamydia today.”* PN4

In later interviews, the change in PN funding with the introduction of the PNIP was seen a facilitator.

*“Because the whole funding for practice nurses has changed that is probably a benefit if you were looking at introducing chlamydia testing because… the practice gets compensated from the government for the hours the nurse works not for who they see in that time frame, so that could actually work to the benefit of nurses doing chlamydia testing.”* PN12

## Discussion

This first qualitative study specifically exploring PNs and chlamydia testing and management in general practice suggests that PNs could take a role in chlamydia testing. The PNs in our study demonstrated support for an increased role in testing and identified important benefits of increased involvement for patients, PNs, GPs, and the community. The PNs raised a number of barriers to PN involvement in testing, but also some practical ways to overcome them.

Issues concerning living in a “small town” were commonly raised by our PNs. It has been suggested that young people accessing local services for sexual health care have fears around confidentiality and anonymity and in small rural towns these problems may be even more acute due to their high visibility and the “inter-connectedness” of the community [23,24]. Possible personal links with health professionals, as highlighted by some of our PNs, may act as a further barrier [23]. However, during a cross-sectional chlamydia prevalence survey carried out as part of ACCEPt, 70% of young people approached agreed to chlamydia testing, with 86% reporting that they were attending their local practice [25]. This suggests that whilst young people in rural towns may not commonly ask for chlamydia testing, they are happy to accept it, if offered. Although PN themselves did not suggest ways to overcome “small town” concerns, the issues could be minimized by PNs reinforcing their duty of confidentiality to young patients, along with normalization of chlamydia testing into routine primary healthcare for this population to reduce stigma [23,26].

GP attitudes towards a PN role in testing were seen as a key barrier by some PNs. Other studies have suggested reluctance among some GPs in Australia in handing over responsibility for aspects of care to PNs, whilst the hierarchical structure and medical dominance that exists in some practices may prevent collaboration between health professionals and limit PNs’ scope of practice [27,28]. However, as has occurred in the UK, the extension of PN roles into areas such as preventative health and screening may alleviate some of the increasing pressures on GPs driven by shortages in workforce and an increase in the burden of disease attributable to chronic conditions [29,30]. In light of this, GPs may therefore find it acceptable for PNs to increase their involvement in chlamydia testing and management. Validation and promotion of the role of the PN in sexual health may also have an effect on the attitudes of GPs and positive work in this area is already being done by state based organisations in Australia [31]. It is also interesting to note there may be some disparity between PNs’ perception of GP attitudes and the actual attitudes of GPs. In a series of qualitative interviews with 44 GPs undertaken as part of ACCEPt, the vast majority supported the concept of PN chlamydia testing, identifying similar benefits to their involvement as were raised by the PNs in this study [32].

PNs raised barriers to their involvement relating to the logistics of testing, such as being unable to order chlamydia investigations for patients without consultation with a GP. As mentioned earlier, most diagnostic pathology investigations undertaken in Australian general practice are claimed through Medicare. Currently, nurse initiated pathology which attracts a Medicare charge is only possible for eligible nurse practitioners and “appropriately qualified and experienced midwives” [33]. Furthermore, the signing of “blank” request forms by GPs, a facilitator suggested by the PNs, or signing *after* a service has taken place is a breach of federal legislation [22], although there is anecdotal evidence that this has happened [34]. It can be argued that in cases where chlamydia testing is clinically indicated, such as for sexually active patients aged 16–29 years, ordering a chlamydia test, independent of immediate GP involvement, is well within the scope of practice of an appropriately trained PN. Systems that permit PNs to initiate chlamydia testing, along with modifications to clinic testing pathways so young people were seen by the PN first, without direct input from a GP, could also address the barrier of time and workload pressures, which was commonly raised and consistent with earlier GP studies on chlamydia testing in the United Kingdom [35,36] and Australia [37,38]. The provision of education and training was a major facilitator for our PNs and the in-depth interviews not only confirmed the importance of the ACCEPt PN training session and education pack as a central component of the ACCEPt PN intervention but also informed its content. The ACCEPt PN education package includes discussion around and suggestions for chlamydia testing pathways that could be used in clinics, along with strategies to employ to minimize time spent and simplify testing consultations. Such strategies include providing “scripts” for PNs to use when offering tests with examples demonstrated for the PNs via DVD [39], checklists to ensure essential elements of the consultation are covered and educational resources for patients to take away.

Funding to cover PN involvement in testing was another identified barrier/facilitator, with a number of PNs suggesting a rebate similar to those previously provided for specific PN tasks such as immunisations. However, as mentioned previously, a number of the interviews took place before the introduction of the new PN funding mechanism, the PNIP. This new arrangement may allow PNs to expand their roles beyond those determined by specific MBS item numbers and include sexual health and chlamydia testing, whilst also addressing the funding for these activities. Resources are available to inform Australian GPs of the rationale and practicalities of PN involvement in sexual health care under the PNIP, and to assist PNs to “make their case” for an expanded role in this area [40]. Formal evaluation of the PNIP is currently being undertaken and aims to identify if the changed funding mechanism has had any impact on the role and function of PNs [41].

The strengths of this study include the recruitment of participants from a diverse range of clinics and from a range of locations across Australia. There are also some limitations. The PNs interviewed reflect those who were selected by their clinics to possibly be involved in chlamydia testing if randomised to the intervention arm of ACCEPt. Thus these PNs may represent a biased sample of PNs who are more “interested” in sexual health. Also, the sample was drawn from clinics in rural and regional areas and some findings such as the issue of ‘small town concerns’ may therefore be context specific.

## Conclusion

Chlamydia infection is an important public health issue for young people and general practice is ideally placed to implement widespread chlamydia testing. However, testing rates must increase to sufficient levels to impact on the burden of chlamydia in this population. As Australia investigates the feasibility, acceptability, efficacy and cost-effectiveness of an organised programme of annual chlamydia testing in general practice and as other countries plan the implementation of screening programmes, our study suggests that PNs could emerge as a potentially valuable resource in contributing to increasing chlamydia testing rates. However, barriers exist that may impede the development of the PN role into this area. Strategies to overcome these barriers and facilitate PN involvement, along with further research into the effectiveness and acceptability of PN chlamydia testing are warranted and should be explored.

## Abbreviations

ACCEPt: Australian control effectiveness pilot
GP: General practitioner
PN: Practice nurse
PNIP: Practice nurse incentive program
RCT: Randomised controlled trial
UK: United Kingdom
US: United States
